# Temporal trends in cardiovascular care: Insights from the COVID-19 pandemic

**DOI:** 10.3389/fcvm.2022.981023

**Published:** 2022-11-08

**Authors:** Matthew Kodsi, Aditya Bhat

**Affiliations:** ^1^Department of Cardiology, Blacktown Hospital, Sydney, NSW, Australia; ^2^School of Public Health and Community Medicine, University of New South Wales, Sydney, NSW, Australia; ^3^School of Medicine, Western Sydney University, Sydney, NSW, Australia

**Keywords:** epidemiology, hospitalization, healthcare, training, COVID-19

## Abstract

In response to the ongoing COVID-19 pandemic, public health care measures have been implemented to limit spread of the contagion and ensure adequate healthcare resource allocation. Correlating with these measures are observed changes in the incidence and outcomes of cardiovascular conditions in the absence of COVID-19 infection. The pandemic has resulted in a reduction in acute coronary syndrome, heart failure and arrhythmia admissions but with worsened outcomes in those diagnosed with these conditions. This is concerning of an underdiagnosis of cardiovascular diseases during the pandemic. Furthermore, cardiovascular services and investigations have decreased to provide healthcare allocation to COVID-19 related services. This threatens an increasing future prevalence of cardiovascular morbidity in healthcare systems that are still adapting to the challenges of a continuing pandemic. Adaption of virtual training and patient care delivery platforms have been shown to be useful, but adequate resources allocation is needed to ensure effectiveness in vulnerable populations.

## The COVID-19 pandemic

**FIGURE 1:**
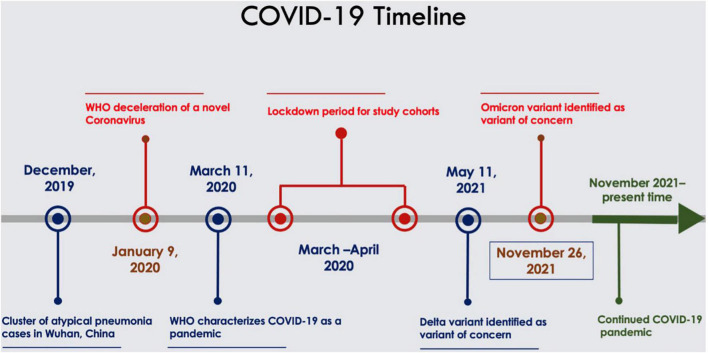
Timeline of COVID-19 pandemic.

In December 2019, a cluster of atypical pneumonia cases were found in Wuhan, China resulting in the World Health Organisation (WHO) declaration of a novel coronavirus on the 9th of January 2020 (1). Subsequently termed coronavirus disease 2019 (COVID-19), worldwide spread of the disease resulted in the WHO declaring a public health emergency of international concern on the 30th of January 2020 and then a pandemic on the 11th of March 2020 (2). High transmissibility of the disease and rapid increases in the number of patients infected resulted in the implementation of public health measures to limit spread. Fearing surging numbers of patients exceeding available resources, *lockdown orders* were initiated in many countries, in particular during March 2020 (Figure 1) (3, 4). After these measures were implemented there were observed decreases in emergency department presentations unrelated to clinical sequelae of the coronavirus disease (5, 6). This trend was also reflected in the field of cardiovascular medicine, with a global decrease in the number of presentations for cardiac disorders reported in observational studies. This raises concerns regarding the current and longer-term effects of COVID-19 on cardiovascular disease presentations and management, which is an evolving area of study given changing dynamics of the disease and the public health response measures. This review aims to identify the trends and the resultant impacts of COVID-19 on the anticipated burden and management of cardiovascular disease in the coming years.

## Impact of COVID-19 on cardiovascular care

### Ischemic heart disease

Ischemic heart disease (IHD) is the number one cause of death globally and was accountable for approximately 16% of deaths worldwide in 2019 (7). In developed countries, annual mortality from IHD had progressively decreased until 2019 (8). Paramount to this improvement is developments in coronary revascularization strategies, early recognition and management of acute coronary syndromes (ACS), as well as improved tertiary prevention strategies in patients with established IHD.

Early reperfusion in ST segment elevation myocardial infarctions (STEMI) has been shown to reduce mortality (9–11) with door-to-balloon and door-to-needle time being used as national standards in guidelines (12). In non-ST segment elevation myocardial infarctions (NSTEMI), revascularization within 72 h is recommended in most patients and is associated with improved clinical outcomes (13). Given these findings, prompt presentation and early intervention in the management of ACS is essential for improved patient outcomes.

Although there was a decrease in the number of ACS presentations in the first 6 months of 2020, the number of deaths from IHD increased in this same period (14). This coincided with the global spread of COVID-19 and associated changes in public health initiatives to curb its spread. Globally, it is estimated that the presentation for ACS decreased between 40% and 50% in the first 6 weeks of initiation of these public health measures in 2020 (15). Observational data demonstrated a greater proportional decrease in the number of NSTEMI presentations when compared to STEMI.

The number of admissions for ACS began to return to levels similar to those seen in pre-pandemic periods several months into the pandemic but were still reduced when compared to previous year control intervals (16). In Europe, there was a 35% decrease in all ACS admissions in England from mid-February until the end of March 2020 (16), a total of 48.4% reduction in ACS in Northern Italy from the 12th to 19th of March 2020 (17) and in Austria there was a 40% reduction in ACS presentations in the last week of March 2020 compared to the first week of March 2020 (18). In the United States, there was a 41% decrease in ACS presentations in March 2020 compared to the same period in 2019 in Boston, MA, USA (19). Gluckman and co-investigators study of six different states reported a 19% decrease in ACS hospitalizations each week from the 23rd of February 2020 until the 29th of March 2020 (20) and in Northern California a reduction of up to 48% in ACS presentations was similarly reported (49% in NSTEMI and 40% in STEMI) (21). Concerningly, despite a decrease in the number of ACS presentations there was an increase in the number of deaths from IHD during the early pandemic period. This increase in deaths from IHD was most pronounced from March 2020 to May 2020 with lower mortality from ACS seen in 2021 (22) suggesting differing trends on these outcomes throughout the pandemic. Whilst a delay in the treatment of STEMI was seen in the early period of the pandemic (23) there is evidence that there was improvement in response times later in the pandemic (24). This demonstrates the effectiveness of adapting health systems to the challenges of the pandemic and the need for modifications in health strategies to minimize the indirect impact of the pandemic on the management of ACS. See Table 1 for a summary on impact of COVID-19 on IHD presentations.

**TABLE 1:** Impact of COVID-19 on ischemic heart disease presentations.

Ischemic heart disease									
**Study**	**Period** **(Pre-pandemic)**	**Period (pandemic)**	**Study design**	**Setting (sites)**	**Study population**	**Patient (n) pre-pandemic**	**Patient (n) post-pandemic**	**Study parameter**	**Findings**
Bhatt et al. (Journal of the American College of Cardiology) (19)	March 2019	March 2020	Cohort Study	Multicenter – Brigham Health System, Boston, MA, USA	Community	144	84	Chest Pain Admissions	Daily decrease in Chest Pain admissions throughout March 2020 –5.5% per day [95% CI: –8.0% –3.0%]
De Filippo et al. (New England Journal of Medicine) (119)	20th February to 31st March 2019	20th February to 31st March 2020	Cohort Study	Multicenter – 15 Hospitals included in Italy	Community	756	547	Acute Coronary Syndromes	IRR 0.70 [95% CI, 0.63–0.78]
						318	248	STEMI Admissions	IRR 0.75 [95% CI 0.64–0.89]
						305	174	NSTEMI Admissions	IRR 0.56 [95% CI 0.46–0.67]
						133	126	UA Admissions	IRR 0.91 [95% CI, 0.72–1.16]
De Rosa et al. (European Heart Journal) (17)	1 week Period March 2019	12–19 March 2020	Cohort Study	Multicenter Study Italy	Community	618	319	AMI Admissions	48.4% decrease [95% CI, 44.6–52.5% (*P* < 0.001)]
						17	31	AMI resulting in death	RR 3.6 [95% CI, 2.0–6.4 (*P* < 0.001)]
						268	197	STEMI Admissions	26.5% Decrease [95%CI, 21.70–32.30% (*p* = 0.0090]
						11	27	STEMI Deaths	RR 3.30 [95% CI, 1.7–6.6, (*p* < 0.001)]
						350	122	NSTEMI Admissions	65.1% decrease [95% CI, 60.30–70.30% (*P* < 0.001)]
						6	4	NSTEMI Deaths	RR 1.9 [95% CI. 0.5–6.7 (*p* = 0.309)]
Gitt et al. (Clinical Research in Cardiology) (120)	1st March – 21st April 2019	1st March – 21st April – 2020	Cohort Study	Single Center – Germany	Community	238	144	ACS Admissions	n.s
						49	46	STEMI Admissions	n.s
						95	50	NSTEMI Admissions	*p* < 0.001
						94	48	UA Admissions	*p* < 0.001
Gluckman et al. (JAMA Cardiology) (20)	30 December 2019 – 22 February 2019	23 February – 29 March 2020	Cohort Study	Multicenter – USA	Community	222	172	Mean Weekly AMI Admission rate	AMI-associated hospitalizations decreased at a rate of –19.0 cases per week (95%CI, –29.0 to –9.0)
						75	57	Mean STEMI Admission rate	
						147	115	Mean NSTEMI Admission rate	
Marfham et al. (Lancet) (16)	1st January to 31st December 2019	23–30 March 2020	Cohort Study	Multicenter – England	Community	3017	1813	Average Weekly admission rate for ACS	Reduction 40%, [95% CI, 37% – 43%]
						2061	1335	Average Weekly admission rate for AMI	Reduction 35% [95% CI, 32–39%]
						621	477	Average weekly admission rate for STEMI	Reduction 23% [95% CI, 16–30%]
						1267	733	Average weekly admission rate for NSTEMI	Reduction 42% [95% CI, 38–46%]
Metzler et al. (European Heart Journal) (18)	2–8 March 2020	23–29 March 2020	Cohort Study	Multicenter – Austria	Community	226	137	ACS Admissions	Decrease in ACS admissions in last week of March 2020 compared to first week of March.
						94	70	STEMI Admissions	
						132	67	NSTEMI Admissions	
Seiffert et al. (Clinical Research in Cardiology) (121)	January – May 2019	January – May 2020	Cohort study	Multicenter study – Germany	Community	3350	2940	STEMI Admissions	12.2% reduction in STEMI admissions (p < 0.05)
						7682	6518	NSTEMI admissions	15.2% reduction in NSTEMI admissions (*p* < 0.05)

Rationalization of this discrepancy in ACS presentations likely involves a change in social, ecological and physical behavior during the early COVID-19 period. During the early stages of the pandemic, fear of nosocomial infection with COVID-19 in hospitals was reported to cause trepidation amongst the general population in presenting to these centers (6, 25). Whilst environmental factors such as decreased air pollution has been proposed as a reason for these trends, this would not fully account for the observation that the decrease in the number of ACS presentations was not as marked later in the pandemic when social distancing measures were still in place (16, 20). Therefore, other issues relating to social distancing and behavioral measures likely contributed to the observed outcomes. The disproportionate decrease in NSTEMI presentations compared to STEMI presentations theoretically may be related to patients in the latter category having more severe and unabating symptoms resulting in prompt presentation (16). This raises the concern of patients with undiagnosed NSTEMI not presenting to hospital during this period which would increase the proportion of this population developing preventable complications such as recurrent myocardial infarction, incident heart failure and fatal cardiac arrhythmia.

The absolute number of percutaneous coronary interventions (PCI) performed decreased when compared to historical control periods (26) but there was an increase in the proportion of NSTEMI patients that underwent inpatient PCI during their hospital stay (16). The cancelation of elective outpatient coronary angiograms, to provide increased resource and personal protective equipment capacity for an anticipated surge in COVID-19 patients, was a significant contributory factor to the overall decrease in the absolute number of coronary angiograms and PCI. The increased frequency of inpatient PCI in NSTEMI may be related to the cancelation of outpatient coronary angiograms, resulting in patients requiring inpatient investigation if invasive coronary assessment was to be performed. Further, early in the pandemic it was identified that there was a higher incidence of patients who met criteria for STEMI who were found to have non-obstructive coronary artery disease on coronary angiography (27). This clinical scenario of faux STEMI presentations has been described in COVID-19 patients (27, 28). Known STEMI mimics including myocarditis, Takotsubo syndrome, pulmonary embolism and diffuse microthrombi are known complications of COVID-19 infection (29–31). There has been an established increase in the proportion of STEMI mimics during the pandemic with a systematic review reporting 19% of all STEMI presentations being found to be mimics during this period (32). Myocarditis in association with COVID-19 is also a well-established condition with elevated cardiac biomarkers and electrocardiography changes often seen, however, its pathogenesis remains poorly understood (33). Further, known cardiovascular risk factors such as hypertension and previously known coronary artery disease, was seen equally in those with true STEMI as well as mimics (32). This presented a unique challenge during the pandemic and may have led to a higher instance of coronary artery assessment to assist in the diagnosis in the setting of raised cardiac enzymes. Those who underwent PCI during admission were observed to have underwent their procedures earlier in the admission with shorter overall length of stay (34). Decrease in length of stay has multiple contributory factors including earlier time to revascularization, fear of COVID-19 contagion in the hospital, and increased demand for hospital beds due to increasing numbers of infected individuals in the community.

This corresponded with decreases in the number of coronary artery bypass grafting (CABG) procedures performed during the same period with multi-center studies in England showing an 80% reduction compared to the weekly average for the year prior (35). It should be noted that guideline approaches to the management of ACS changed during this period where patients that would previously be considered suitable for CABG may be managed with PCI given the decreased capacity of cardiac intensive care due to presentations from COVID-19 (36). Cardiothoracic surgery places operators at risk of transmission of COVID-19 from infected individuals given the use of intraoperative transoesophageal echocardiography, risk of air leak with thoracic procedures, chest drain insertion into the pleural cavity and cardiopulmonary bypass (37). Therefore, concerns of nosocomial infections to surgical staff as well as increased demand for limited hospital resources may, in part, explain this observed trend.

### Heart failure

Heart failure hospitalizations were also seen to decrease following the implementation of social distancing and public health measures. This has been reported in observational data in North America (19, 38, 39) and Europe (40–43) (Table 2) with some centers reporting up to a 17% decrease in heart failure presentations when compared over the same period in 2019. The initial decline in presentations coincided with the initial public health measures with admissions approaching pre-COVID 19 rates later into the pandemic period. Despite decreases in admissions, those that required hospitalization were demonstrated to have worse outcomes with higher rates of in-hospital mortality (41, 42, 44). Despite worse inpatient mortality rates, the absolute number of deaths secondary to heart failure was not significantly greater than pre-pandemic control periods. To rationalize this finding, it has been speculated that, unlike patients with end-stage heart failure, those with mild exacerbations were more likely to defer hospital presentation during COVID-19. This phenomenon has been demonstrated with ACS and stroke, with patients delaying presentation to hospital (45) during this period which could also be theoretically inferred with heart failure. Furthermore, social isolation was demonstrated to have reduced the incidence of non-COVID-19 communicable diseases such as influenza, which has shown association with heart failure exacerbations (46). Unfortunately, many of the available studies detailing heart failure outcomes during the COVID-19 period used percentage of admitted patients dying from heart failure as primary outcomes and not absolute number of overall heart failure deaths. This limits the generalization of the claim that heart failure deaths overall remained the same during the pandemic. The reduction in heart failure admissions was most profound during the early pandemic period with a rebound in admissions in the later 2020 period that was similar to the pre-pandemic period (44). This emphasizes the impact of “lockdown” measures, particularly in the initial pandemic period on hospital presentation. Patient clinical characteristics, including New York Health Association functional class, of those admitted with heart failure were similar in the early pandemic period and the first 2 months of 2021 (47). However, the proportion of patients initiated on angiotensin receptor neprilysin inhibitor during hospital stay was higher in the later period. The cause of this finding is not certain but propensity for escalation of heart failure treatment in an inpatient setting given the disruptions in outpatient services may have contributed. The study by Cannata et al. reported that a greater proportion of heart failure patients were managed on general medical wards during the pandemic (44). Reduced availability of cardiology beds during the pandemic and redeployment of cardiac staff may partially explain this trend. This is of particular concern given evidence that those with heart failure managed by dedicated cardiology teams has been shown to improve inpatient 30-day mortality rates (48).

**TABLE 2:** Impact of COVID-19 on heart failure presentations.

Heart failure									
**Study**	**Period** **(Pre-pandemic)**	**Period (pandemic)**	**Study design**	**Setting (sites)**	**Study population**	**Patient (*n*) pre-pandemic**	**Patient (*n*) post-pandemic**	**Study parameter**	**Findings**
Andersson et al. (Circulation: Heart Failure) (40)	12–31 March 2019	12–31 March 20202	Cohort Study	Multicenter – Denmark	Community	720	398	New Onset Heart Failure	Age and sex adjusted IRR in pandemic period 0.69 [95% CI, 0.63–0.77]
						353	215	Hospitalization for Worsening Heart Failure	Age and sex adjusted IRR in pandemic period 0.70 [95% CI, 0.61–0.80]
Bhatt et al. (Journal of the American College of Cardiology) (19)	March 2019	March 2020	Cohort Study	Multicenter – Boston, MA, USA	Community	197	91	Hospitalization for Worsening Heart Failure	Decline in Hospitalizations rates for Heart Failure HF –5.8% per day [95% CI: –8.3% to –3.3% (*p* ≤ 0.004)
Bollmann et al. (European Society of Cardiology Heart Failure) (41)	13 March – 30 April 2019	13 March – 30 April 2020	Cohort Study	Multicenter – Germany	Community	2782	1979	Number of Heart Failure Admissions	IRR of Heart Failure admissions per day during pandemic compared to 2019 period 0.71 [95% CI, 0.67–0.76 (*p* < 0.01)]
						154	138	In Hospital Mortality for Heart Failure	In-hospital mortality 5.5% during pre-pandemic period, 7.0% during pandemic period, (*p* < 0.05). OR 0.78 [95% CI, 0.61–0.99 (*p* = 0.04)
Cannatà et al. (European Journal of Heart Failure) (44)	7 January – 14 June 2019	7 January – 14 June 2020	Cohort Study	2 Center Study-London, UK	Community	794	578	Number of Heart Failure Admissions	Decrease in HF admissions during pandemic period compared to pre-pandemic (*p* < 0.001) Increased in-hospital mortality in 2020 compared with 2019 (*p* = 0.015)
Doolub et al. (European Society of Cardiology Heart Failure) (42)	7 January – 2 March 2020	3 March – 27 April 2020	Cohort Study	Single Center Study – UK	Observational	164	119	In-hospital referrals to Heart Failure Team	27% reduction in Heart Failure referrals (*p* = 0.06)
						18	25	30 Day Mortality Rate of Patients referred to Heart Failure team	21% increase in risk of 30 Days inpatient mortality during pandemic period (Risk ratio = 1.9, [95% CI, 1.09–3.3])

### Cardiac arrhythmia

The number of admissions for cardiac arrythmias, including atrial fibrillation (AF), decreased during the early period of the COVID-19 pandemic (Table 3) (43). Observational data in Europe has reported decreases in the number of new onset as well as total AF admissions (49–51). Of concern, one study suggested the average duration of symptomatic AF prior to presentation to hospital increased during the pandemic when compared to earlier control periods (52). This study excluded those without a definite timing of symptom onset related to AF in determining duration until presentation and therefore these findings cannot be applied to the significant proportion of patients with asymptomatic AF. Another trend observed was that the population of patients with newly diagnosed AF on average had a lower CHA_2_DS_2_-VASc score when compared to preceding control periods (50). Despite this decrease in number of AF presentations, there is evidence of increased AF burden in those with implantable cardiac devices during the pandemic when compared to an earlier control period (53). Decreases in the number of AF diagnoses, despite increased AF burden in those with devices, projects concern that decreased presentations during the pandemic may be leading to under diagnosis and inadequate treatment. This threatens risk of preventable complications from AF, most notably ischemic stroke. Despite this trend, the number of ischemic stroke admissions was seen to decrease globally, with several centers reporting decreases of up to 35% (54, 55). This is an unexpected finding given the association between COVID-19 and thromboembolic phenomenon such as stroke. This trend has been associated with a decrease in the number of transient ischemic attack (TIA) presentations as well as those with mild stroke symptoms (54, 56). Of concern, despite a decrease in stroke presentations there was an observed increase in stroke mortality during the early pandemic period (57, 58). These findings may, in part, be explained by a variation in methodology of stroke diagnosis across the aforementioned studies. Gabet et al. (57) included patients discharged from hospital who had stroke as the main diagnosis during hospital stay whilst the stroke population included by Sharma et al. (58) were patients in whom the initial emergency service primary provider impression was stroke. Methodology for determining stroke-related mortality also differed, with death certification with diagnosis of cerebrovascular event and death during hospital admission for stroke being used in different studies. Discrepancy between stroke admissions and deaths highlights concerns that that the actual incidence of stroke may not have significantly decreased but that less patients are presenting for medical care. Underdiagnosis of stroke inherently results in decreased early treatment in this population which therefore increases the risk of further stroke, disability, and death (59). In conjunction with poorer patient outcomes, this will inherently result in increased future economic and physical burden on health systems already stressed with the demand of healthcare with COVID-19.

**TABLE 3:** Impact of COVID-19 on arrhythmia presentations.

Arrhythmia									
**Study**	**Period (pandemic)**	**Pre-pandemic**	**Study design**	**Setting (sites)**	**Study population**	**Event (*n*) -pandemic**	**Event (*n*) pre-pandemic**	**Study parameter**	**Findings**
König et al. (Clinical Cardiology) (43)	13 March – 10 September 2020	15th March to 12th September 2019	Cohort Study	Multicenter – Germany	Community	14286	16971	Inpatient Admissions for Arrythmia	16% decrease [95% CI, 15–17% decrease, (*p* < 0.001)]
O’Shea et al. (Europace) (53)	21 January to 29 April 2020	21 January to 29 April 2019	Cohort Study	Multicenter – USA	Community – 10,436 Patients with pacemaker or AICD	2722	2209	AF episodes > 6 min	IIR 1.33, 95% CI 1.25 – 140, *p* < 0.13
						1544	1099	AF Episodes > 1 h	IRR 1.65, 95% CI 1.53-1.79, *p* < 0.001
						707	521	AF Episodes > 6 h	IRR 1.65, 95% CI 1.53 – 1.79, *p* < 0.001
O’Shea et al. (European Heart Journal) (64)	21 January to 29 April 2019	21 January to April 2019	Cohort Study	Multicenter USA	Community – 1719 Patients with Cardiac Device	349	331	Number of Patients with Ventricular Arrhythmia	OR 0.87, 95% CI 0.67–1.12, *p* = 0.28
						3988	5346	Number of Ventricular Arrhythmia Episodes	IRR 0.69, 95% CI 0.56–0.85 m -<0001
Holt et al. (European Heart Journal) (50)	12 March – 1 April 2020	12 March 2019 – 1 April 2019	Cohort Study	Multicenter, Denmark	Community	562	1053	New-onset AF	47% decrease
	12–18 March 2020	12–18 March 2019				232	352		IRR 0.66 [95% CI, 0.56–0.78]
	19–25 March 2020	19–25 March 2019				182	340		IRR 0.53 [95% CI, 0.45–0.64]
	26 March – 1 April 2020	26 March – 1 April 2019				148	361		IRR 0.41 [95% CI, 0.34–0.50]
Ueberham et al. (European Heart Journal – Quality of Care and Clinical Outcomes) (51)	13 March – 11 April 2020	15 March – 13 April 2020	Cohort Study	Multicenter, Germany	Community	44.4	77.5	Discharge Diagnosis of AF per Day	IRR 0.57 [95% CI 0.54 – 0.61 (*p* < 0.01)]
	12 April – 16 July 2020	14 April – 18 July 2019				59.1	63.5		IRR 0.93 [95% CI 0.90 – 0.96 (*p* < 0.01)]
Bilaszewski et al. (International Journal of Environmental Research and Public Health) (52)	July – December 2020	July – December 2020	Cohort Study	2 Center, Poland	Community	213	292	Number of patients Treated for AF	Not provided
						10 h (4–48)	5.5 h (3–23)	Mean time from AF onset to ED presentation	Not provided

Ventricular arrythmias present a substantial risk of sudden death. Major world events and natural disasters have been associated with temporal increases in ventricular arrhythmias and sudden cardiac death (60, 61). These trends have been attributed to increased emotional and physical stress, which has been shown to increase the incidence of ventricular arrhythmias (62, 63). Despite presenting many of the societal impacts and stress seen in other disasters, a cohort study of patients with implantable cardiac devices found a decrease in ventricular arrhythmia burden during the pandemic (64). A clear explanation for this finding is unclear. Factors that are speculated to have contributed to a decrease in ventricular arrhythmias in this cohort are the protracted course of the pandemic differing from the finite time course of other historical disasters that were associated with increased ventricular arrythmias. Other postulated causes for this decrease during the pandemic are reduction in occupational exposures and physical stress from working at home, but this has not been proven. Incongruent to this finding, is the widely reported increase in out of hospital cardiac arrests during the pandemic (65–67). It remains uncertain whether this increased in sudden cardiac deaths is related directly to COVID-19 or indirect effects of the virus delaying patient presentation to emergency services.

**FIGURE 2:**
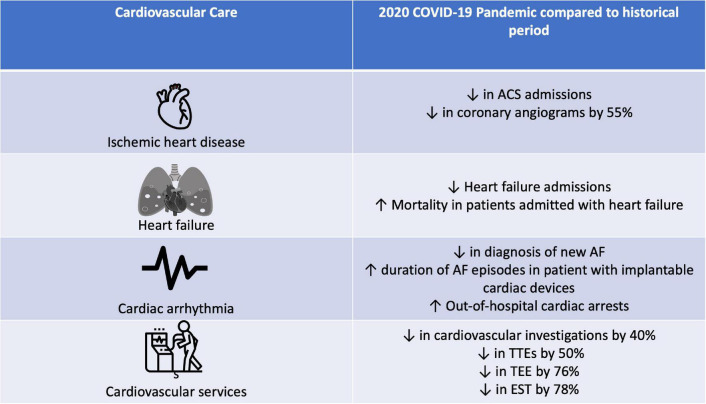
Impact of COVID-19 pandemic on cardiovascular admissions.

A pictorial summary of the impact of the COVID-19 pandemic on cardiovascular admissions is shown in Figure 2.

### Hypertension

Hypertension is estimated to affect 1.4 billion patients globally and 46% of adults in the USA (68, 69). Sustained blood pressure control in patients with hypertension has demonstrated a reduction in cardiovascular events (70). The COVID-19 pandemic has resulted in disruptions in previously established models of care delivery in hypertension. Natural disasters have previously been demonstrated to be associated with reductions in adequate blood pressure control in hypertensive patients (71). This has, in part, been attributed to temporal interruptions in access to health care services. Reported trends in the management of hypertension during the COVID-19 pandemic has varied. Laffin et al. found in an observational study of 464585 participants that there was an increase in blood pressure in participants between April and December 2020 with mean changes in systolic blood pressure each month of the pandemic period (April–December 2020) of 1.1–2.5 mmHg (72). Furthermore, during the pandemic period more patients with hypertension were found to have moved into a higher category of hypertension as defined by the 2017 American Heart Association guidelines (72, 73). In contrast this same study did not find differences in blood pressure between 2019 and January–March 2020. Conflicting to these findings are the findings in Brazil (74), Italy (75), and France (76) that did not demonstrate worsening blood pressure control during the early period of the pandemic. In the study by Feitosa et al. those that here already on pharmacological therapy for hypertension were found to have improved blood pressure readings in the early period of the pandemic (74). Whilst the assessed impacts of hypertension care during the pandemic has varied there remains concerns regarding the long term impacts of the pandemic on this condition. During the pandemic period there were reduction in the assessment of hypertension in the primary care setting (77). The pandemic has also been associated with increased weight gain (78) and sedentary lifestyle (79) which are risk factors known to contribute to hypertension. Further assessment of long-term trends in hypertensive care are required to determine the long term impacts of the pandemic on this condition.

### Cardiac investigations

In the context of profound changes in hospital operations to address the increased demand for medical personal and resources, there have been observed changes in cardiac procedures. Globally, there was a decrease by over 40% in cardiac diagnostic procedures in March 2020 when compared to the same period in 2019 with even greater declines seen in April 2020 (80). The global peak reductions compared to March 2019 were 59% in transthoracic echocardiography, 76% in transesophageal echocardiography, 78% in exercise stress testing and 55% in coronary angiography. Low-, middle-, and high-income countries were all impacted with the former two groups being disproportionately affected.

In a study of a UK district hospital there was a 43% decrease in the number of invasive coronary angiography, 63% reduction in computed tomography coronary angiography, and cancelations of all transesophageal echocardiograms, transcatheter aortic valve implantation and electrophysiological studies (81). This study also gave insight into triaging systems during the early pandemic regarding cardiac devices, with permanent pacemaker (PPM) insertions for third- and second-degree heart blocks remaining uninterrupted but implantation for symptomatic bradycardia or sinoatrial disease often being delayed with a 50% overall reduction in PPM insertion.

Electrophysiological studies (82) as well as catheter ablations for atrial (83) and ventricular arrythmias have all reported a decline in procedures, with decreases of over 90% reported in England during the early pandemic period (84). Widespread decreases in the number of cardiac devices inserted have been reported, including rates of automatic implantable cardioverter defibrillators (AICD) for primary and secondary prevention (85).

### Cardiovascular training and research

The redistribution of resources, community uncertainty and changes in models of care poses great challenges in cardiology training, research and outpatient management. The aforementioned decreases in cardiac procedures and investigations threatens the quality of training in cardiology. Decreases in invasive and non-invasive procedures impacts trainee exposure and therefore proficiency in these skills. Furthermore, redistribution of staff to meet emergent needs in patient care has been observed in many centers (86) during outbreaks of COVID-19 which has limited trainee exposure to dedicated cardiology services. This, in conjunction with the delays in patient presentations, threatens delays in diagnosis of cardiac conditions which may result in increased long-term morbidity and will likely require increased demand for cardiology services in the future to manage these patients. Delayed presentations of cardiovascular conditions risks increased mortality within the community as well as growing populations of patients with heart failure, strokes and arrhythmias. Given the increased demand on healthcare systems with the COVID-19 pandemic, the addition of a growing number of patients with potentially preventable cardiovascular morbidity would further strain these institutions.

Cardiac rehabilitation programs were also temporarily suspended in many areas to assist in limiting spread of disease. This has led to the implementation of home cardiac rehabilitation programs which faces similar challenges to telehealth but also has the potential of expanding availability of these programs, particularly to geographically isolated individuals (87). Research and further developments in cardiology have also been impacted by the pandemic with fear of contagion impacting patient participation in health centers (88). Paramount to the running of clinical trials is ensuring patient safety which has been complicated by the risk of transmission of COVID-19 in health care sites. The increased number of infected patients requiring treatment in healthcare centers during the pandemic has limited the safety of these facilities in following up trial participants. Furthermore, many clinical trials are targeted toward treatment of patients that are most vulnerable to complications from COVID-19 infection, which led to concerns regarding the safety of frequent visits to health care facilities. This posed a great disruption to the frequency of participant follow up in clinical trials and impacts on the depth of data collected during study periods. This has impacted the amount of research performed with estimated decreases in non-COVID-19 trials of up to 80% during the early pandemic period (88). Reallocation of trial resources toward management and prevention of COVID-19 have also contributed to declines in trials not associated with COVID-19, with cancelation of many existing research projects to meet the demands of the pandemic (89). Economic strain from the pandemic has also resulted in many universities reducing the number of postdoctoral researchers (90).

**FIGURE 3:**
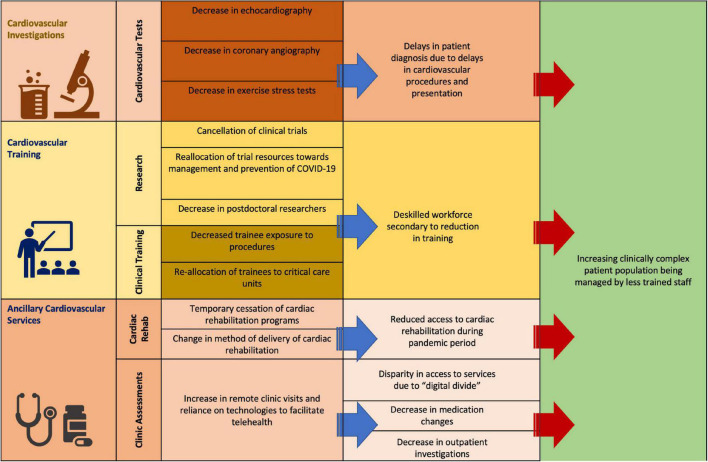
Impact of COVID-19 on cardiovascular services.

In addition to the impacts on cardiovascular research, training in cardiology has changed greatly in response to COVID-19. Negative impacts of the pandemic on cardiovascular research and training, in conjunction with the potential of increasing cardiac morbidity from delayed presentations, poses a great threat to future outcomes in patients with cardiovascular conditions. This may result in a “perfect storm” of an increasing population of more clinically complex patients whose treatment is impeded by stunted advances in cardiovascular medicine and lack of adequately trained staff. Figure 3 provides a pictorial summary of the impact of COVID-19 on cardiovascular investigations, training, and services.

## Long COVID

Compounding the concerns of growing cardiovascular morbidity during the pandemic and post pandemic period is the growing healthcare burden of “long COVID.” Upto a third of patients with COVID-19 report symptoms following resolution of infection (91). Cardiovascular concerns in the post-infectious period include long term complications from cardiac injury during COVID-19 infection as well as persistent symptoms following acute viral infection. Cardiac complications from acute infection have been well documented including arrythmia (92), myocarditis (93), and increased risk of myocardial ischemia (94) with resulting complications from these syndromes contributing to further cardiovascular morbidity during the pandemic. Left ventricular dysfunction following COVID-19 associated myocardial injury has been documented (95–97). Viral infections, including respiratory viruses (98), have been seen to be linked to atherosclerotic disease (99, 100). This has been seen in the setting in COVID-19 infection (101, 102) and threatens increasing cardiovascular morbidity. Concerningly, there is evidence of acceleration of cardiovascular disease in the setting of COVID-19 with increased 1 year risk of IHD, heart failure, myocarditis and arrhythmias being seen following infection, even in those not requiring hospitalization (103). Vaccination has shown promise in mitigating these trends, with improvement in the mortality of STEMI patients in a population in 2021 when vaccination was available when compared to the 2020 period when vaccination was not available. Furthermore, in this population those that were vaccinated had a lower risk of in-hospital mortality when compared to unvaccinated individuals in the same time period (104). In those with long COVID, cardiac symptoms such as chest pain, dyspnea and palpitations have been well documented. Cardiac MRI in a proportion of these patients have shown evidence of myocardial edema, fibrosis and impairment of ventricular function (105). Long-term outcomes in those with cardiovascular complications during and following infection remains uncertain and is the subject of further investigation.

### Pathways through the pandemic

With the continued spread of COVID-19 and emergence of variants, the impacts of the pandemic are projected to continue (106, 107). Therefore, without appropriate adaptions of healthcare systems, the observed trends of delayed presentations with risk of growing mortality and morbidity will continue. Supporting this concern has been the observation of a rebound increase in heart failure admissions following lockdown periods (40). Fear of contagion has impeded patient confidence in presenting to hospital in ACS and other acute cardiovascular conditions. Given the disproportionate decrease in NSTEMI and unstable angina presentations, those with milder symptoms may not appreciate the urgency of presentation. Therefore, community education regarding the need to seek medical review, even in the absence of severe symptoms, is paramount. Further community education regarding the precautions and protocols used in healthcare systems to minimize risk of contracting COVID-19 in hospital may also reduce patient hesitancy toward hospital presentation.

To limit pathogen exposure to staff and outpatients, many outpatient clinics have shifted to a telehealth model of care (108). Whilst useful in managing contagion spread, this has limitations. Naturally, this form of care limits the ability to examine patients, identify cardiac murmurs to guide further investigation and manually monitor vital signs that are intrinsic in not only management of cardiovascular risk factors but also titration of many cardiac medications. Reviews in the implications of telehealth services in the delivery of care have found significant changes in clinical practice compared to in-person visits prior to the pandemic. This included reductions in the proportion of appointments that resulted in medication changes or diagnostic investigation during the pandemic period in those that had video and telephone consultations, with voice only visits being the most affected (109). In addition, utilization of new technologies has met resistance in widespread implementation from both physicians and patients, at least in some part related to the complexity of adopting new health care models (110). Appropriate implementation of such technologies requires appropriate funding and support staff to effectively manage some of the most vulnerable populations, particularly the elderly and frail (111). Furthermore, financially disadvantaged individuals or those in lower income countries may lack the resources required in order to implement this model of care (111). This “digital divide” threatens to cause further disparity in the delivery of healthcare if not considered in the modeling of outpatient services in the pandemic and post-pandemic period.

Diagnosis of cardiac arrythmia in the outpatient setting during the pandemic has proven challenging, with a decrease in health care contact and the reliance on telehealth impacting the accessibility of electrocardiograms. Practice updates have been published to address this issue with the utilization of direct-to-consumer electrocardiogram devices and prescribed ambulatory rhythm monitors, but this has limitations to widespread use including cost, need for expansion of infrastructure in health systems and patient education (112). Given profound and rapid changes in health care during the pandemic, changes in the traditional approach to managing disease requires appropriate education of physicians and patients. Dedicated teams to implement and promote new technologies for widespread implementation is essential to not only assist uptake of new technologies in the general population but also to ensure appropriate access to those that may have difficulty in utilizing such technologies.

Given the concerns of increasing cardiovascular morbidity secondary to patients deferring presentation during the pandemic, optimal training of staff and resource allocation to cardiology is essential. The described impacts of the pandemic on cardiology trainee exposure and therefore proficiency are likely to continue whilst physical distancing measures remain in place. Simulation training in cardiology has been an area of growing interest in recent years and provides promise as a method of developing procedural skills whilst limiting risk of infection to staff (113). Furthermore, in order to address reduced trainee exposure to procedures during the pandemic, virtual training provides a pathway for skill developmental to ensure competence of future cardiologists. Simulation-based procedural training has been used for transesophageal echocardiography (114, 115) and coronary angiography (116), and has been demonstrated to improve technical proficiency in trainees and enhance education beyond lecture-based learning. Whilst such methods pose promise, the impacts of COVID-19 on cardiology are far reaching and adaptions to training requires large-scale implantation. This would require appropriate funding to ensure accessibility amongst trainees, including the cost of establishing the appropriate infrastructure in training centers to support these technologies. The growing opportunity cost from reduction in cardiovascular research also threatens to stagnate further developments and improvements in outcomes in a growing population of patients with cardiac disease. Appropriate financial and technical support is required to allow cardiac research to continue in the dynamic circumstances of the pandemic.

**FIGURE 4:**
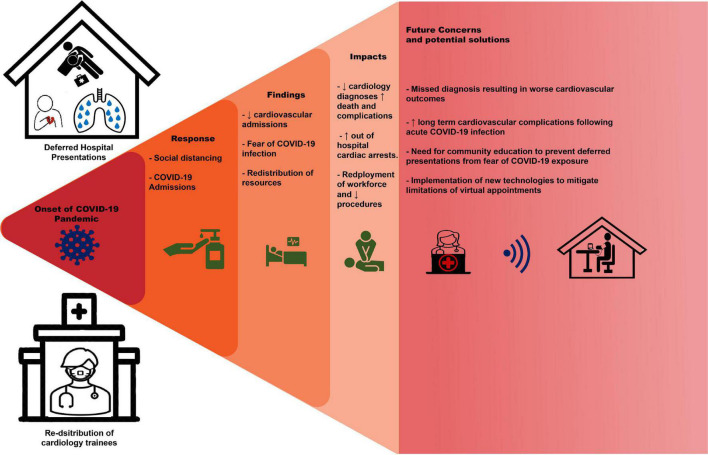
Trends in cardiovascular care during COVID-19 pandemic and proposed solutions.

Telemedicine has not only been shown to provide a roadmap to management paradigms in acute cardiovascular disease, establish cardiac rehabilitation and chronic disease programs but also explore primary prevention options (117). Similarly, numerous articles have already stated the importance of environmental pollution as a significant contributor to overall cardiovascular morbidity and mortality (118). In the broader context of the current pandemic, the current findings of reduced all-cause cardiovascular hospitalizations, has allowed for derivation of important pathways for clinicians, in order to reduce overall patient morbidity and mortality. Figure 4 provides a pictorial summary of trends in cardiovascular care during the COVID-19 pandemic and proposed solutions.

## Conclusion

There has been a significant reduction in all-cause cardiovascular hospitalizations during the COVID-19 pandemic, consistent with the rapid establishment of social containment measures worldwide. Subsequently, there were observed reductions in cardiovascular procedures and significant interruptions to training. These trends were most apparent in the early pandemic period and correlated with increased cardiovascular mortality. Given the potential impacts of increasing cardiovascular morbidity on healthcare systems focused upon management of COVID-19, action is required to minimize these indirect impacts of the pandemic on cardiovascular services.

## Author contributions

AB and MK contributed to conception and design of the study and conducted the data collection. MK wrote the first draft of the manuscript. AB contributed to sections of the manuscript. MK created the figures with review by AB. Both authors contributed to manuscript revision, read, and approved the submitted version.
